# TNFR/TNF-α signaling pathway regulates apoptosis of alveolar macrophages in coal workers' pneumoconiosis

**DOI:** 10.18632/oncotarget.18921

**Published:** 2017-07-01

**Authors:** Qing-Zeng Qian, Xiang-Ke Cao, Hai-Yan Liu, Guo-Ying Zheng, Qing-Qiang Qian, Fu-Hai Shen

**Affiliations:** ^1^ School of Public Health, North China University of Science and Technology, Tangshan 063000, P.R. China; ^2^ College of Life Sciences, North China University of Science and Technology, Tangshan 063000, P.R. China; ^3^ Department of Neurology, Tangshan Gongren Hospital Affiliated to North China University of Science and Technology, Tangshan 063000, P.R. China

**Keywords:** coal mixture workers, coal workers' pneumoconiosis, cumulative dust exposure, alveolar macrophages, apoptosis

## Abstract

We explored the role of TNFR/TNF-α signalingin apoptosis among alveolar macrophages (AM) and its relevance to the development of coal workers’ pneumoconiosis (CWP). Purified alveolar macrophages (AMs) were prepared from bronchoalveolar lavage fluid harvested from 366 CWP patients and 120 healthy subjects enrolled inthe study. The purified AMs were then divided into control, SOD, anti-TNFR, TNFR and NFkB inhibitor groups and analyzed for apoptosis usingflow cytometry (sub-diploid peak) and western blotting (Bcl-2, Caspase-3 and Caspase-8 expression). We found thatAM apoptosis washigher amongCWP patients than thehealthycontrols. Expression ofBcl-2, Caspase-3 and Caspase-8 was higher inAMs from CWP patientsthan in those from the controlsand correlated with increased AM apoptosis. Univariate and multivariate analyses suggested that CWP grade, initial exposure time, exposure time inyears, and CWP onset agewereall associated with altered levels of Bcl-2, Caspase-3 and Caspase-8. Inhibition of TNFR/TNF-α signaling usinganti-TNFR antibody, SOD or NFkB inhibitionreduced AM apoptosisand decreased Bcl-2, Caspase-3 and Caspase-8 expression. These data suggestinhibition of a TNFR/TNF-α signaling pathway is a potentiallyeffective means ofalleviating CWP by inhibiting AM apoptosis.

## INTRODUCTION

Although there is increased emphasis on physical health and regularhealth examinations at work places, occupational disease rates have increased significantlywithrapid development of China’s coal industry [1–3]. Long-term exposure of mining workers to coal dustcontributes to increased risk of chronic occupational lung diseases and irreversible lung damage [4, 5, 6]. Coal workers’ pneumoconiosis (CWP) is one of the most common lung diseasesassociated with different degrees of pulmonary function abnormalities in coal mining workers [7]. It is one of the most serious occupational diseases in Chinathatresults inlungfibrosis due to long-term inhalation of coal dust [8, 9]. Previous studies have associatedlong-term exposure to coal mine dust with CWP mortality [10]. Stringent enforcement of occupational exposure limits for coal mine dust has resulted in declined prevalence of CWP among underground coal miners [11]. However, miners still working incontemporary conditionscontinue to develop lung diseases, therebyimplyingthat further restrictions and regulations in occupational dust exposure are necessary [12].

Apoptosis refers toa genetically controlled program of cell death that is involved in elimination of old or unhealthy cells that could be detrimental to organismal health. In recent years, investigation of CWP pathogenesis has revealed thatapoptosis ofalveolar macrophages (AM) is involved in development of pneumonia and pulmonary fibrosis [13]. The three major signal transduction pathwaysthat regulate AM apoptosis includethe mitochondrial pathway, the endoplasmic reticulum pathway, and the membrane-associated cell death receptor pathway [14–17]. Thecell death receptorsbelonging to the members of the TNFR super-family initiate apoptosis upon binding to their ligands and operate mainly through three basic signal transduction pathwaysnamely, Fas/Fas ligand (FasL), TNFR/TNF-α, and TRAILR/TRAIL [18, 19, 20]. These pathways ultimately modulate critical players of apoptosis includinginitiator and executioner caspases, P53, Bcl-2 and other apoptotic regulatory proteins [21]. Therefore, blocking the death receptor mediated apoptosis of AM is probably an effective measure to prevent or cure CWP [22]. Therefore, in this study, weexplored the role of TNFR/TNF-α signaling pathway in AM apoptosis and association to CWP. We also investigated if inhibition of TNFR/TNF-α signalingcould provide a therapeutic basis for the early prevention and treatment of CWP.

## RESULTS

### Baseline characteristics of CWP patients

In the exposed group, the mean age of the 366 coal mine workers was 47.8 ± 9.2years (range: 24 to 65 y) and the mean body weight of included subjects was 57.7 ± 10.3 kg (range:42∼86 kg). In the control group, the mean age of the 120 coal mine workers was 48.2 ± 8.4 years (range:28 to 68 years) and the mean body weight of control subjects was 56.8 ± 10.5 kg (range:43∼85 kg). As shown, there were no significant differences between the exposed and the control group with regard to mean age and body weight (both *P* > 0.05). The other baseline characteristics includingsmoking status, initial working time, occupational history, current occupation, retirement period and others were all similar between the exposed and the control groups (all *P* > 0.05; Table 1).

**Table 1:** Comparisons in smoking rate, pulmonary function and pulmonary function indexes between the case group and the control group

Group	Exposed group (*n* = 366)	Control group (*n* = 120)	χ^2^/*t*	*P*
Age (year)	47.80 ± 9.20 (24∼65)	48.20 ± 8.40 (28∼68)	0.422	0.673
Weight (kg)	57.70 ± 10.30 (42∼86)	56.80 ± 10.50 (43∼85)	0.409	0.827
Smoking distribution			0.462	0.645
Non-smoking	165 (45.08%)	57 (47,50%)		
Smoking	201 (54.92%)	63 (52.50%)		
Initial working time	21.10 ± 4.16	22.00 ± 5.50	1.890	0.059
Work years	23.50 ± 5.85	24.34 ± 6.78	1.311	0.191
Retirement period	3.83 ± 1.11	4.05 ± 1.06	1.905	0.057

Note: Initial working time, the starting age of workers exposed to dust; work years, the length of the time employed; retirement period, the duration after stopping dust exposure of workers exposed to dust.

### Apoptosis status of AM in CWP patients

**Figure 1:**
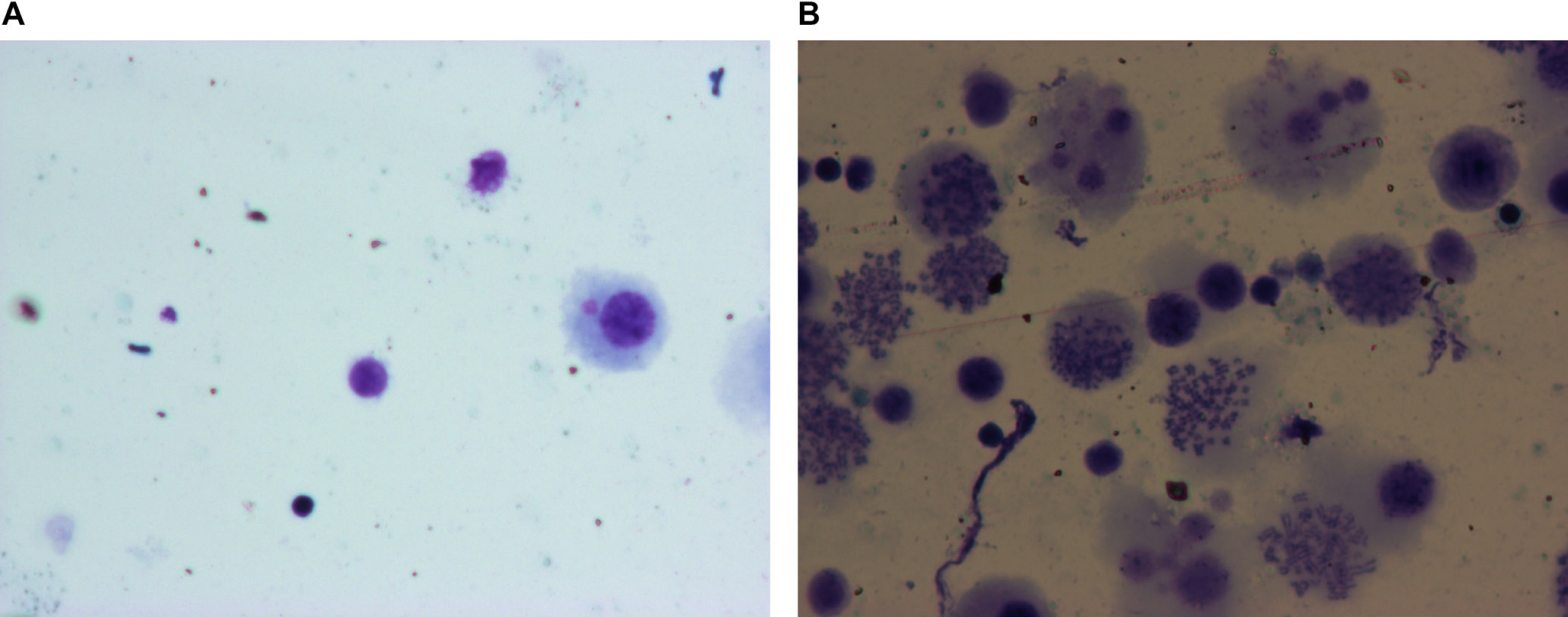
Representative H&E stained photographs of alveolar macrophages undergoing apoptosis as observed underlight microscope (1000×) (**A**) Alveolar macrophages from normal controls; (**B**) Apoptotic alveolar macrophages from CWP patients.

Figure 1 shows therepresentative images of control and CWP patient alveolar macrophages (AM). The mature normal AM from control subjects showed increased cell volume, integral cell membrane, round or oval shaped cell-centric nucleusand were free of intracellular dust particles. The apoptotic AM cells from CWP patients showed marginalizedchromatinandfragmented nucleus with massive apoptotic bodies typical ofapoptosis.

### Comparison of AM apoptosis among different subgroups of CWP patients

**Figure 2:**
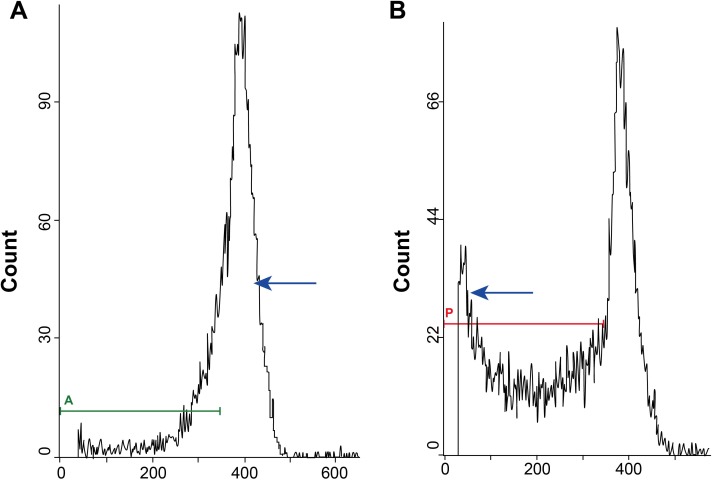
Flow cytometry analysis of apoptoticalveolar macrophages cells showing the sub-diploid (sub-G0/G1) peak (**A**) FACS plots showing normal peaks forcontrol alveolar macrophages; (**B**) FACS plot showing sub-diploidpeak representing alveolar macrophages undergoing apoptosis.

The apoptotic AM cells were analyzed by flow cytometry and the sub-diploid peak representing the AM cells undergoing apoptosis is shown in Figure 2. Our analysis showed that AM cells from CWP stage I and stage II patient groups showed significantly higher apoptosisthan those from normal controls (both *P* < 0.05). Further, apoptosis in AM cells from CWP stage I patients was significantlyenhanced compared to thosefromCWP stage IIpatients (*P* < 0.05). Table 2 shows the apoptotic index ofsubjects in the exposed group stratified by age, smoking, initial exposure time, exposureyears, and CWP onset age. Except for the subgroup representing different exposure years (*P* < 0.05), there was no significantdifferencein apoptosis betweenothersubgroups (all *P* > 0.05).

**Table 2:** Comparisons of alveolar macrophages apoptosis among different subgroups based on age, smoking, initial working time, occupational time, and working age for occurrence in the exposed group

Group	N	Apoptotic index	χ^2^/*t* or *F*	*P*
Age			0.597	0.551
< 40	156	27.22 ± 5.47		
≥ 40	210	26.88 ± 5.25		
Smoking (duration time, years)				
0	165	26.52 ± 5.40	1.876	0.155
< 30	88	26.28 ± 5.07		
≥ 30	113	27.62 ± 5.89		
Initial working time (years)			0.791	0.429
< 20	144	26.69 ± 5.44		
≥ 20	222	27.18 ± 6.00		
Work years (years)			12.290	< 0.001
< 20	170	23.02 ± 5.12		
≥ 20	196	29.98 ± 6.12		
Working age for occurrence			1.403	0.161
< 10	130	27.33 ± 5.85		
≥ 10	136	26.48 ± 5.48		

### Analysis of the expression of key apoptotic regulatory proteins in AM from CWP patients

As shown in Table 3, we observed thatthe expression of key apoptotic regulatory proteins, Bcl-2, Caspase-3 and Caspase-8 was significantly enhancedin the exposed group compared to those in the control group (all *P* < 0.05). Besides, there were positive correlation between elevated expression of Bcl-2, Caspase-3 and Caspase-8 and enhanced AM apoptosis (all *P* < 0.05).

**Table 3:** Comparisons of the expression levels of Bcl-2, Caspase-3 and Caspase-8 in the exposed group

Group	Case group	Control group	χ^2^/*t*	*P*
Bcl-2	0.291 ± 0.044	0.211 ± 0.038	17.850	< 0.001
Caspase-3	0.338 ± 0.050	0.267 ± 0.040	14.140	< 0.001
Caspase-8	0.237 ± 0.041	0.219 ± 0.034	3.137	0.002

Note: these results were not influenced following the adjustment for cofounders such as age, smoking history and other baseline factors.

Furthermore, we conducted stratified analysesinvestigating the association between the expression levels of Bcl-2, Caspase-3 and Caspase-8 with factors likeage, smoking, CWP stages, initial exposure time, exposureyears, and CWP onset age (Table 4). Our data showed significant association between the expression levels of Bcl-2, Caspase-3 and Caspase-8 anddifferent subgroups based onage, smoking, initial exposure time, exposureyears, and CWP onset age (all *P* < 0.05). In addition, multivariate analysis indicated that altered expression levels ofBcl-2, Caspase-3 and Caspase-8significantly correlated with CWP stages. Besides, initial exposure time, exposureyears, and CWP onset agecorrelated withthe expression levels of Caspase-3. Also, Caspase-8 expression levels were associated withexposureyears, whereas expression levels of Bcl-2 were influenced by CWP onset age (all *P* < 0.05).

**Table 4:** Comparisons of the expression levels of Bcl-2, Caspase-3 and Caspase-8 among different subgroups based on age, smoking, initial working time, occupational time, and working age for occurrence in the exposed group

Group	N	Bcl-2	Caspase-3	Caspase-8
Age				
< 40	156	0.356 ± 0.047	0.354 ± 0.047	0.032 ± 0.045
≥ 40	210	0.310 ± 0.035^*^	0.322 ± 0.042^*^	0.038 ± 0.043
Smoking (duration time, years)				
0	165	0.345 ± 0.054	0.401 ± 0.054	0.376 ± 0.050
< 30	88	0.336 ± 0.048	0.367 ± 0.041^*^	0.382 ± 0.056
≥ 30	113	0.342 ± 0.043	0.375 ± 0.050^*^	0.367 ± 0.048
Initial working time (years)				
< 20	144	0.288 ± 0.043	0.376 ± 0.055	0.256 ± 0.035
≥ 20	222	0.231 ± 0.034^*^	0.345 ± 0.036^*^	0.218 ± 0.040^*^
Working years (years)				
< 20	170	0.288 ± 0.060	0.377 ± 0.062	0.236 ± 0.056
≥ 20	196	0.243 ± 0.051^*^	0.332 ± 0.054^*^	0.228 ± 0.047
Working age for occurrence				
< 10	130	0.227 ± 0.057	0.376 ± 0.067	0.321 ± 0.046
≥ 10	136	0.234 ± 0.048	0.337 ± 0.068^*^	0.327 ± 0.050

Note: ^*^P < 0.05 when compared between two subgroups.

### Regulatory role of TNFR/TNF-α signal pathway in AM apoptosis from CWP patients

Next, we investigated the role of TNFR/TNF-αsignaling pathway in regulating AM apoptosis. We observed that the apoptotic index of AM in the SOD group (14.23 ± 2.01) was significantly lower than that in the control group (23.47 ± 2.68) and the TNFR group (18.33 ± 1.80). Furthermore, the apoptotic index of AM in the anti-TNFR group (14.00 ± 1.45) and NF-kB inhibitor group (13.52 ± 1.77) were also significantly reduced when compared to the TNFR group (all *P* < 0.05).

**Figure 3:**
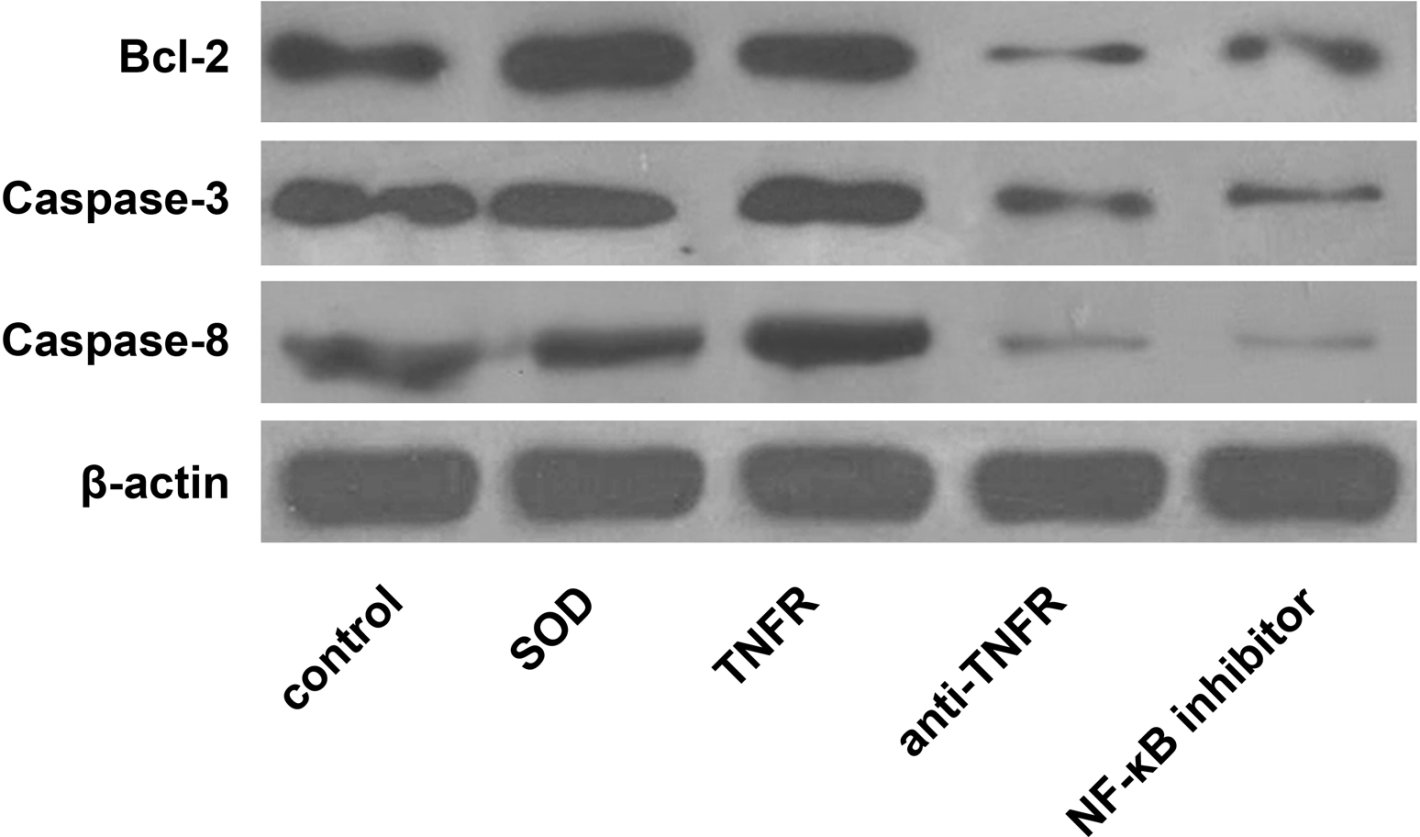
Western blot analyses of apoptosisregulatoryproteins among the 5 experimental groups of alveolar macrophages The 5 experimental groups are as follows: A, control group; B, SOD group; C, TNFR group; D, anti-TNFR group; E, NF-kB inhibitor group.

Consequently, the expression levelsof Bcl-2, Caspase-3 and Caspase-8 were significantly decreased when TNFR/TNF-α signaling pathwaywas blocked SOD and was least compared to all other 4 groups (all *P* < 0.05). In addition, blocking theTNFR/TNF-α signal pathwaywithanti-TNFR antibody or NFkB inhibitor significantly reduced expression levels of Bcl-2, Caspase-3 and Caspase-8 compared with the control group and the TNFR group (all *P* < 0.05). Thedata areshown in Figure 3 and Table 5.

**Table 5:** Comparisons of the expression levels of Bcl-2, Caspase-3 and Caspase-8 among different subgroups based on the application of different intervention approaches

Protein	Group	SOD treatment	Group	anti-TNFR treatment	Group	NF-kB inhibitor treatment
Bcl-2						
	A	0.291 ± 0.056	A	0.324 ± 0.045	A	0.350 ± 0.044
	B	0.147 ± 0.034^*^	C	0.264 ± 0.035^*^	C	0.265 ± 0.040^*^
	C	0.245 ± 0.034^**^	D	0.160 ± 0.033^**^	E	0.165 ± 0.031^**^
Caspase-3						
	A	0.198 ± 0.024	A	0.231 ± 0.050	A	0.231 ± 0.041
	B	0.134 ± 0.030^*^	C	0.224 ± 0.042	C	0.221 ± 0.038
	C	0.200 ± 0.037^**^	D	0.154 ± 0.032^**^	E	0.143 ± 0.030^**^
Caspase-8						
	A	0.234 ± 0.050	A	0.238 ± 0.056	A	0.242 ± 0.042
	B	0.153 ± 0.034^*^	C	0.230 ± 0.043	C	0.195 ± 0.034^*^
	C	0.213 ± 0.042^**^	D	0.182 ± 0.040^**^	E	0.162 ± 0.027^**^

Note: A, control group; B, SOD group; C, TNFR group; D, anti-TNFR group; E, NF-kB inhibitor group. ^*^P < 0.05 when compared with group A; ^**^P < 0.05 when compared with group B or group C.

## DISCUSSION

CWP is a result of long-term inhalation of coal mine and silica dust thatresults in impaired pulmonary function and lung diseases, therebyrepresenting a relevant occupational hazard for coal miners [8]. Despite dust control and reduction technology being utilized in the recent decades, the morbidity of CWPhas showedincreased incidence [23]. Sincethe mechanistic details of CWP pathogenesis are not clear, the prevention and treatment of CWP has been a challenge. Hence, it is very important to explore mechanismsthat promote CWP in order to obtain greater understanding of this disease and identify effective treatment methods. *In vivo* and *in vitro* studies have shown that AM plays an important role in CWP [24, 25]. Thedust particles that enter the alveoli are phagocytosed by the AM cells that results in their activation and caspase-dependent apoptosis. In the present study, we investigated the role of TNFR/TNF-αsignaling pathway in the pathogenesis of CWP and explored if it represented a relevant strategy to inhibit CWP.

Apoptosisis a systematic program of cell deathwhich is initiated by specific signaling pathways in response to either external or intracellular stimuli and executed by sequential activation of specific apoptosis regulatory proteins like caspases [26, 27]. Cellular apoptosis is animportant physiological mechanism in multicellular organisms, which is critical for maintaining body’s normal development and homeostasis and is involved in organ development, tissue repair and immune regulation [28, 29]. In the present study, we observed that alveolar macrophages showed typical characteristics of apoptosis likechromatin marginalization and nuclear fragmentation into massive intracellular apoptotic bodies [30]. Our study indicated that AM cells from CWP patients were prone to excessive apoptosis than that of the normal controls. Although mechanisms regulating CWP are not clear, AM apoptosis has been postulated to be responsible for the pathological development of pulmonary fibrosis [31]. Specifically, apoptosis of AM cells results in production of large amounts of inflammatory cytokines and fibrogenic factors thatpotentiallyplay an important role in the pathogenesis and development of CWP [32]. Apoptosisincludessignal transduction, sequential activation of apoptotic genes, execution of apoptosis and subsequent removal of apoptotic cells [33]. We demonstrated that the AM cells from CWP patients underwent excessive apoptosis than controls as visualized by the sub-diploid (sub-G0/G1) apoptosis peak in flow cytometry assays.

*In vivo* and *in vitro* studies have shown that AM plays an important role in CWP. AM arethe main target cells exposed to dust andincreased AM numbers are observed in CWP patients that correspondto excess amount of dust in the lungs [34]. Length of exposure to dust and the onset age are critical determinant factors in the incidence of CWP. Our study demonstrated that the apoptosis index of AM increased with the increase degree of CWP. Further, our data showed that increased length of exposure to dust in the CWP patients resulted in higher expression levels of Bcl-2, Caspase-3 and Caspase-8. Hence, our data suggested that longer coal dust exposure timeresulted in higher levels of AM apoptosis, which in turn increased incidence of CWP.

More importantly, we observed the relationship between TNFR/TNF-α signal transduction pathway on AM apoptosis [35] and the higher expression of critical apoptotic proteins in CWP patients, namelyBcl-2, Caspase-3 and Caspase-8. Our data also showed that blocking TNFR/TNF-α mediated AM apoptosis by anti-TNFR antibody resulted in decreased apoptosis and expression of Bcl-2, Caspase-3 and Caspase-8, thereby suggesting that TNFR/TNF-α signal transduction pathway was a key player in AM apoptosis and CWP.

Caspases area group of cysteine-aspartate proteases that are activated in a step wise manner upon apoptotic signaling [36]. The apoptotic signaling pathways activateinitiatorCaspasessuch asCaspases-8, -9, and -10, which then cleave and activate theeffectorCaspases such as Caspases-3, -6, and -7 that cleave critical proteins required for cellular homeostasis and function resulting eventually in deathof target cells [37–39]. Caspase-8 is an initiator caspase found upstream of the Caspase cascade, whereasCaspase-3 is an important effector caspasethat is critical for accomplishing cellular apoptosis [40]. We demonstrated that treating AM cells with anti-TNFRantibody that blocks TNFR/TNF-αsignaling apoptotic pathway resulted in decreased apoptosis of AM cells and diminished expression of Bcl-2, Caspase-3 and Caspase-8. This suggested that inhibitors ofTNFR/TNF-αsignaling may potentially benefitCWP patients therapeutically as it would enhance survival of AM cells and thereby alleviate CWP

In conclusion, our study demonstrates that constant and excessive exposure to coal dust results in excessive apoptosis of AM due to stimulation of the TNFR/TNF-αapoptotic signaling pathway that leads to enhanced expression of apoptosis related proteins like Bcl-2, Caspase-3 and Caspase-8. Our study also demonstrated that inhibiting TNFR/TNF-αsignaling pathway byeither anti-TNFR antibody or inhibitors like SOD or NFkB inhibitors results in reduced AM apoptosis suggesting a potential therapeutic target to alleviate CWP.

## MATERIALS AND METHODS

### Ethical statement

The study was approved by the Ethics committee ofTangshan Gongren Hospital affiliated to North China University of Science and Technology. All subjects or their legal guardians provided written informed consents. Study protocols followed the ethical principles for medical research involving human subjects according to the Helsinki Declaration [41].

### Study subjects

For this study, 366 coal mine workersfrom Kailuan Colliery, China (exposed group)that received treatment in Tangshan Gongren Hospital affiliated to North China University of Science and Technologywere recruitedas the research subjects. They were diagnosedfor pneumoconiosis in accordance with the specific standards of *National Diagnostic Criteria of Pneumoconiosis* [42] and subdivided into CWP stage I (*n* = 180) and CWP stage II (*n* = 186) groups. Subjectswere enrolled if (1) they were Han males; (2) they were exposed todustover 1 year; (3) they hadcomplete physical examination during the past two years; and (4) had complete records ofoccupational history or could be supplemented via checking working files. Patients with related lung diseases such as pneumonia, lung cancer, and active pulmonary tuberculosis were all excluded from this study. Also, patients diagnosed with pneumoconiosis combined with pulmonary complications, severe heart&lung diseases and infectious diseases were also excluded. In addition, 120 coal mine workers without previous history of anypulmonary diseases were enrolled in the present study as the control group.

Information regarding baseline characteristics was collected by face to facequestionnaires to avoid contradictions or difficulties in obtaining complete information or through telephone interviews with retired workers. The data included (1) demographic characteristicslike age, date of birth, gender, weight and height, smoking status, initial exposure time, occupational history, current occupation, and retirement age; (2) dust exposure history, which included initial exposure time, average exposure time, and age of dust removal; (3) pulmonary disease history that included time of initial diagnosis, grade, complications and onset time of complications. Most of the information was obtained from the medical records of the patients from Tangshan Gongren Hospital affiliated to North China University of Science and Technology. Further information wasobtained from occupational health examination records and face to face inquiries. Information regarding dust exposure, diagnostic information of pneumoconiosis and other relevant complications wascollected from the hospital recording files atTangshan Gongren Hospital affiliated to North China University of Science and Technology.

### Experimental methods

#### Alveolar macrophage cell cultureand grouping

The subjects enrolled for this study underwent large capacity double lung simultaneous irrigation operation under general anesthesia at the Pneumoconiosis rehabilitation center of National Coal Mine Safety Supervision Bureau. After an appropriate amount of bronchoalveolar lavage fluid washarvested, it wasfilteredthroughthree layers of sterile gauze, followed by centrifugation. The supernatant was discarded and the cell pelletswere subsequently washedthrice in PBSand centrifuged to obtain the AM cell suspension that was diluted in appropriate amounts 10% DMEM medium and cell counts obtained.

The AM cell suspension (5 × 10^6^) was seeded in 6-well culture plates in 2ml DMEM mediumcontaining10% heat inactivated FBS and placed in a culture box with 84% humidity, 5%CO_2_and 37°C for 2 h. The non-adherent cells were discarded and fresh DMEM medium was added to the adherent cells that represented the purified AM.

After purification, the AM cells were divided into five experimental groups as follows: (1) control group without any treatment; (2) SOD group that received200U/ml SOD; (3) TNFR group that received 50ng/ml anti-TNF-α antibody; (4) anti-TNFR group that received 50ng/ml anti-TNF-α antibody with200ng/ml anti-TNFR; and (5) NFkB inhibitor group.

Furthermore, as for AM culture and harvest, after reagents were added, the cells were incubatedfor 24 h (84% humidity, 5% CO_2_ volume fraction in 37°C). The 5 groups of cells were grown for 24 h at 37°C and 5% CO_2_ following which the supernatant was discarded and the adherent cells were trypsinized with 0.25% trypsin for 1 min. Then, the cells in each group were collected, and washed thrice with PBS followedby centrifugation for 10 mins at 1000rpm and the pelleted cells were resuspended and counted. The collected cells were stored for analyzingapoptosis. A portion of the cells were stained with hematoxylin&eosinand observed under the microscope to assess morphological changes associated with apoptosis.

### Flow cytometry assay for AM apoptosis detection

The AM cells in the 5 experimental groups were fixed with cold 70% ethanol and analyzed in a flow cytometer, among which the reaction tube was added with 200 μl binding buffer and 5 μl propidium iodide. The percent cells in the sub-G0/G1 peak represented cells undergoing apoptosis. The sub-G0/G1 was identified from the FSC-H versus SSC-H plots.

### Western blot analysis of apoptotic signaling proteins

The AM cells from the 5 experimental groups were incubated in protein lysis buffer (BeyotimeInstitute of Biotechnology) for 30 min at 4°C with intermittent vigorous mixing. After centrifugation at 1000 rpm for 15 min at 4°C, the supernatant was stored at –80°C. The protein amount in the lysate was quantified using the Bradford assay kit (BioRad, Hercules, CA, USA). Then, the protein samples were separated on 10% SDS PAGE and transferred onto PVDF membrane (1.5 h). The initial voltage was 60 V, and elevated at 120 V when the front edge of the bromophenol blue into the seperation gel. the After blocking, the membrane was incubated with primary antibody (Anti-human Bcl-2, Caspase-3 and Caspase-8 and anti-human β-actin, dilution ratio of 1:2000, provided by the Santa cruz co., Ltd, CA, USA) for 1hfollowed by incubation with 1:2000dilutedgoat anti-rabbit secondary antibody for 45 min at 37°C. The blot was developed with ECL method (ECL reaction mixture, Santa cruz co., Ltd, CA, USA) and the protein bands were quantified by the image analysis software IPP 6.0.

### Statistical analysis

SPSS17.0 software was used for statistical analysis. Continuous variables were presented as mean ± S.D; categorical variables were presented as frequencies and percentages. Comparisons between continuous variables were analyzedby *t* test and *F* test, whereas Wilcoxon rank-sum test was used for comparisons of non-randomlydistributed continuous variables. The chi-square test was used for categorical variables. Correlations were analyzed by Pearson correlation analysis. A *P* value of < 0.05 was considered statistically significant.
